# Barriers and Facilitators in Access to Diabetes, Hypertension, and Dyslipidemia Medicines: A Scoping Review

**DOI:** 10.3389/phrs.2022.1604796

**Published:** 2022-09-02

**Authors:** Carla Castillo-Laborde, Macarena Hirmas-Adauy, Isabel Matute, Anita Jasmen, Oscar Urrejola, Xaviera Molina, Camila Awad, Catalina Frey-Moreno, Sofia Pumarino-Lira, Fernando Descalzi-Rojas, Tomás José Ruiz, Barbara Plass

**Affiliations:** ^1^ Centro de Epidemiología y Políticas de Salud, Facultad de Medicina Clínica Alemana, Universidad del Desarrollo, Santiago, Chile; ^2^ Biblioteca Biomédica, Facultad de Medicina Clínica Alemana, Universidad del Desarrollo, Santiago, Chile; ^3^ Carrera de Medicina, Facultad de Medicina Clínica Alemana, Universidad del Desarrollo, Santiago, Chile

**Keywords:** access, diabetes, barriers, facilitators, non-communicable chronic diseases (NCDs), hypertension (HTN), dyslipidemia, medicines

## Abstract

**Objective:** Identify barriers and facilitators in access to medicines for diabetes, hypertension, and dyslipidemia, considering patient, health provider, and health system perspectives.

**Methods:** Scoping review based on Joanna Briggs methodology. The search considered PubMed, Cochrane Library, CINAHL, Academic Search Ultimate, Web of Science, SciELO Citation Index, and grey literature. Two researchers conducted screening and eligibility phases. Data were thematically analyzed.

**Results:** The review included 219 documents. Diabetes was the most studied condition; most of the evidence comes from patients and the United States. Affordability and availability of medicines were the most reported dimension and specific barrier respectively, both cross-cutting concerns. Among high- and middle-income countries, identified barriers were cost of medicines, accompaniment by professionals, long distances to facilities, and cultural aspects; cost of transportation emerges in low-income settings. Facilitators reported were financial accessibility, trained health workers, medicines closer to communities, and patients’ education.

**Conclusion:** Barriers and facilitators are determined by socioeconomic and cultural conditions, highlighting the role of health systems in regulatory and policy context (assuring financial coverage and free medicines); providers’ role bringing medicines closer; and patients’ health education and disease management.

## Introduction

Cardiovascular diseases remain the first cause of death worldwide, representing 32% of all global deaths in 2019 [1]. Conditions such as hypertension [2], diabetes [3], and dyslipidemia [4] are among the most prevalent risk factors for their development.

The treatment of these three conditions provides a convenient entry point to control other non-communicable diseases (NCDs) [5]. They share commonalities in delivery of services and model of care (e.g., continuous monitoring, multidisciplinary treatment team, importance of diet), also share the importance of pharmacotherapy [6, 7] and achieving patient adherence to control them [8].

Equitable access to medicines for these risk factors is crucial to reduce the burden of NCD [9, 10]. For instance, providing medicines at primary care level [11] has shown as an effective approach to reduce blood-pressure-related NCDs deaths. Moreover, the universal health coverage Sustainable Development Goal requires access to safe, adequate quality, and affordable medicines [12, 13]. However, even when low-cost and generic drugs are available to treat NCDs, many patients cannot access [10].

Access to health care in general has been defined as “the opportunity to reach and obtain appropriate health care services in situations of perceived need for care” [14]. Following this framework, Koh et al. [7] frame access to medicines as the interaction of accessibility and ability (to perceive, seek, reach, pay, engage), in a process that goes from medication need to medication utilization. In long-term therapies, access to appropriate medicines affects patients’ adherence [7], which is key for successfully treating these types of conditions [15].

Access to medicines is a complex concept that includes the dimensions of availability, accessibility, accommodation, affordability, and acceptability [16, 17]. They can become barriers or facilitators, challenging the health system’s capacity, and hindering people’s ability to obtain the medication they need to maintain or improve their health [18]. Because these barriers are complex and interconnected, access to medicines should be tackled from a health system perspective, considering access constraints at different levels (individual, household and community; health service delivery; health sector; public policies cutting across sectors; and international and regional level), and both demand- and supply-side constraints [19].

Health policy would benefit from systematic information on the scope of common and specific barriers and facilitators in access to medicines for treating diabetes, hypertension, and dyslipidemia. Isolated evidence exists about barriers and facilitators faced by patients or from the perspective of health providers, but no evidence was identified that systematizes the findings [20–29]. In addition, no current or ongoing scoping or systematic reviews on the topic were identified. Considering the importance of these three conditions in the global disease burden, updated evidence that systematizes the various findings related to access to medicines to treat such conditions is essential to visualize the entire phenomenon comprehensively, to guide articulated strategies and effective health solutions.

The objective of this scoping review is to comprehensively map and summarize knowledge related to the identification of barriers and facilitators in access to medicines, worldwide, for diabetes, hypertension, and dyslipidemia, considering patient, health care provider, and health system perspectives.

## Methods

The scoping review was conducted following the Joanna Briggs Institute (JBI) Guidance for conducting systematic scoping reviews [30] and reported according to the Preferred Reporting Items for Systematic reviews and Meta-Analyses extension for Scoping Reviews (PRISMA-ScR) [31].

### Review Question

What are the barriers and facilitators in access to medicines for the treatment of diabetes, hypertension, and dyslipidemia from the perspectives of patients, health care providers, and the health system?

### Inclusion Criteria

**FIGURE 1:**
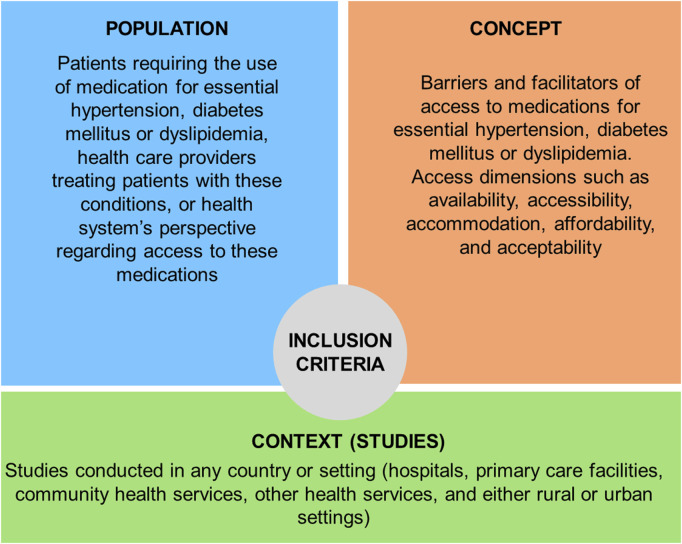
Inclusion criteria based on PCC method for scoping reviews (Chile, 2022).

The inclusion criteria were based on the components of the Population, Concept, and Context (PCC) method of JBI [30]. See Figure 1.

#### Population

Focused on patients requiring medication for essential diabetes mellitus, hypertension, or dyslipidemia, health care providers treating patients with these conditions, or the health system’s perspective (health systems factors, interventions, policies, or programs) regarding access to these medications.

#### Concept

Barriers or facilitators to access to medicines for the treatment of essential diabetes mellitus, hypertension, or dyslipidemia were reviewed. Under the conceptual frameworks by Tanahashi [16], and Penchansky and Thomas [17], the access dimensions included were: availability, accessibility, accommodation, affordability, and acceptability. Studies related to recreational drugs and medications in animals were excluded since these topics are far from the focus of the study. Furthermore, adherence to medications was not included because patient adherence is related to disease management rather than to access to medicines.

#### Context

Studies conducted in any country or setting, including hospitals, primary care facilities, community health services, other health services, and either rural or urban settings.

### Data Sources and Search Strategy

The following biomedical databases were consulted: PubMed (NCBI), Cochrane Library (free access by Ministry of Health, Chile), CINAHL Plus with Full Text (EBSCO), Academic Search Ultimate (EBSCO), Web of Science (Clarivate), and SciELO Citation Index (Clarivate). Grey literature was searched in the OpenGrey database and through homepages of the following international organizations: World Health Organization (WHO), Pan American Health Organization (PAHO), and the Organization for Economic Co-operation and Development (OECD). A hand search was also performed in the World Bank databases.

This review considered all types of scientific articles and grey literature (manuals, technical papers, scientific conferences reports, recommendations, reports, and policy briefs).

To identify relevant studies, an experienced biomedical librarian (AJS) and two researchers trained in public health/epidemiology (MH and OU) searched the literature based on the stages recommended by the JBI [32]. Firstly, an initial limited pilot study search was conducted in PubMed (MH and AJS) using keywords and Medical Subject Headings (MeSH) terms associated with “drugs,” “access barriers,” “access facilitators,” “diabetes,” “arterial hypertension,” “dyslipidemia,” “hypercholesterolemia,” and “metabolic syndrome.” This search strategy was conducted in the other databases, verifying the consistency of results to the research question. Secondly, the keywords identified in the relevant articles of the initial search were used to develop the complete planned search strategy (see Appendix).

A language filter was applied to exclude all studies or documents available in languages other than English, French, Portuguese, and Spanish, and only studies in humans were included. There was no restriction of publication year considered. Finally, a hand search of reference lists of relevant articles identified in the final search was conducted (Reference review or Backward citation).

The information search strategy for the full review was carried out on 27 January 2020, in the case of indexed literature, and 28 February, 12 March, and 29 April 2020, for grey literature.

Following the search, reference management software (Mendeley) was used to download and manage the reference database. Additionally, the search results were managed using Microsoft Excel, by creating separate sheets for the various stages of the search and review process.

The titles and abstracts resulting from the search (identification phase) were independently screened by two reviewers from the research team (CC-L, IM, MH-A, OU, XM, CA, CF-M, SP-L, FD-R, TR, and BP), selecting full-text articles or reports for the eligibility phase, in which the texts were reviewed by two independent reviewers. Disagreement, in any of these two phases, was resolved by consulting a third reviewer. It should be noted that five of the reviewers have extensive experience in public health research and epidemiology, as well as experience in literature reviews.

Once the titles and abstracts to be included were selected, the full text was searched and obtained through free access, requested from the head of the Biomedical Library of our University (AJS), or purchased. The entire selection process was diagrammed in a flow chart identifying each stage (identification, screening, and eligibility), the number of results and eliminations at each stage, and the reason for their exclusion.

### Data Extraction

Relevant data extraction from selected studies was performed according to Peters et al. [32]. The research team piloted and executed the extraction process. The pilot allowed the researchers to test the designed matrix and adjust it according to the feedback obtained from this phase. In addition, it allowed the criteria to be standardized for the final data extraction. Once the extraction was completed, three researchers (CC-L, IM, MH-A) conducted a review of this stage, validating each of the barriers and facilitators extracted from the documents and adjusting when necessary.

Data were compiled in a single spreadsheet, containing the articles’ main characteristics, along with specific aspects of the research question of this review. Data extraction categories included: author(s), date, title, journal, volume, issue, pages, year of publication, country, aims/purpose, study population and sample size, methodology/methods, intervention type, comparison, outcomes, and key findings that relate to this scoping review question.

### Analysis and Presentation of Results

Data from the selected studies and documents were synthesized by three researchers (CC-L, IM, MH-A) through an iterative process, according to the research question.

To summarize the data, descriptive statistics were calculated on the general aspects of the documents and on the barriers and facilitators identified. Also, a qualitative description and an analysis of these barriers and facilitators was included. Finally, the data characterization enabled gaps in knowledge to be identified [33].

## Results

### Overview

**FIGURE 2:**
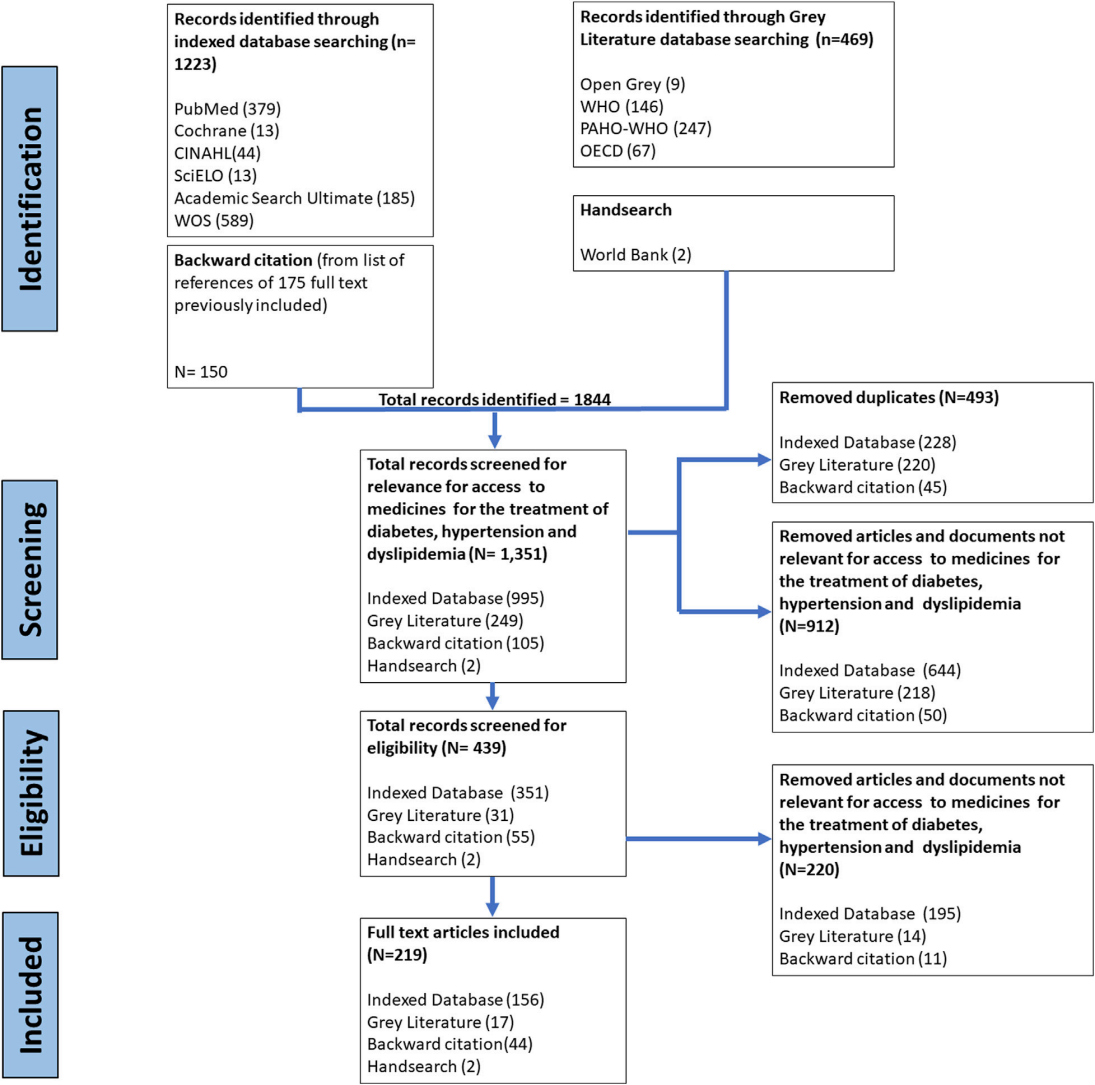
Flow chart of study selection for scoping review process (Chile, 2022). WOS, Web of Science; WHO, World Health Organization; PAHO, Pan American Health Organization; OECD, Organization for Economic Co-operation and Development.

The search identified 1,844 studies, from which 219 records were finally included in the scoping review (198 indexed literature and 30 grey literature) (Figure 2).

Of the total publications with a study design (189 indexed literature, 1 grey literature), 32.1% were cross-sectional (*n* = 61), qualitative (*n* = 47, 24.7%), “report, review or discussion article” (16.3%, *n* = 31), cohort (6.3%, *n* = 12), mixed method (5.8%, *n* = 11) and literature review (4.7%, *n* = 9) (Table 1). Nine of the 30 selected grey literature documents (30%) were country reports, while eight and six were technical and regional reports respectively (26.7% and 20%), and the remaining documents were policy report, recommendations (10%, *n* = 3), and a PhD thesis (3.3%, *n* = 1).

**TABLE 1:** Summary of characteristics of the reviewed documents (Chile, 2022).

Characteristics of included documents (*n* = 219)		
Characteristic	*n*	%
Publication year		
<2000	2	0.9
2000–2004	12	5.5
2005–2009	45	20.5
2010–2014	61	27.8
2015–2020	99	45.2
Publication type		
Indexed database	189	86.3
Grey literature	30	13.6
Country report	9	30.0
Technical report	8	26.7
Regional report	6	20.0
Policy report	3	10.0
Recommendation	3	10.0
PhD thesis	1	3.3
Study design (*n* = 190)		
Cross sectional	61	32.1
Qualitative study	47	24.7
Report, review or discussion article	31	16.3
Cohort study	12	6.3
Mixed method	11	5.8
Literature review	9	4.7
Other^a^	5	2.6
Systematic literature review	3	1.6
Experimental design	2	1.1
Longitudinal and observational study	2	1.1
Systematic literature review and meta- analysis	2	1.1
Cohort and cross sectional	1	0.5
Quasi-experimental design	1	0.5
Experimental design (rationale and design)	1	0.5
Prospective study with survey	1	0.5
Qualitative study (multi-method)	1	0.5
Study population		
Patients	97	44.3
Patients and Health providers	34	15.5
Patients, Health providers, Health system	32	14.6
Health system	25	11.4
Health providers	16	7.3
Health providers and Health system	7	3.2
Studied diseases		
Diabetes	105	47.95
Diabetes and hypertension	48	21.92
Hypertension	39	17.8
Diabetes, hypertension, dyslipidemia	19	8.67
Dyslipidemia	6	2.73
Non-communicable diseases	2	0.91

a Indexed articles that could not be classified in the previous designs, such as the elaboration of recommendations or the development of a roadmap. Source: based on [4, 7, 9, 15, 16, 22–24, 26, 27, 35–245].

### Participants

Based on the PCC model, the target populations of the 219 publications were: 44.3% patients only, 15.5% patients and health care providers, 14.6% patients, providers and the health care system, 11.4% only health system, 7.3% only health care providers, 3.2% health system and providers together. Regarding the conditions studied, of 219 results, 78.5% addressed diabetes, 48.4% hypertension and 11.4% dyslipidemia (Table 1).

### Context

The studied countries classified by income and region respectively, according to the World Bank classification [34], are presented in Supplementary Tables S2, S3. Out of the 219 publications, 68% could be classified by income: 38% (*n* = 84) in the context of high-income countries (HIC), 13% (*n* = 29) in upper middle-income economies (UMIC), 14% in lower middle-income (LMIC), and 2% low-income (LIC). The most studied region was North America (31.5%) with 69 publications, of which the United States (US) had 60 and Canada nine records. Representing Latin America and the Caribbean (6.8%, *n* = 15), Brazil leads with six (2.7%) documents.

The first publication identified was an article from 1986, while the year with the highest number of publications was 2018 (*n* = 24) (Table 1).

### Concept: Barriers and Facilitators in Access to Diabetes, Hypertension, and Dyslipidemia Medicines

The classification of barriers and facilitators presented below emerges from the in-depth review of the texts and the findings found in the review; and is based on the theoretical models of Tanahashi [16], and Penchansky and Thomas [17].

**FIGURE 3:**
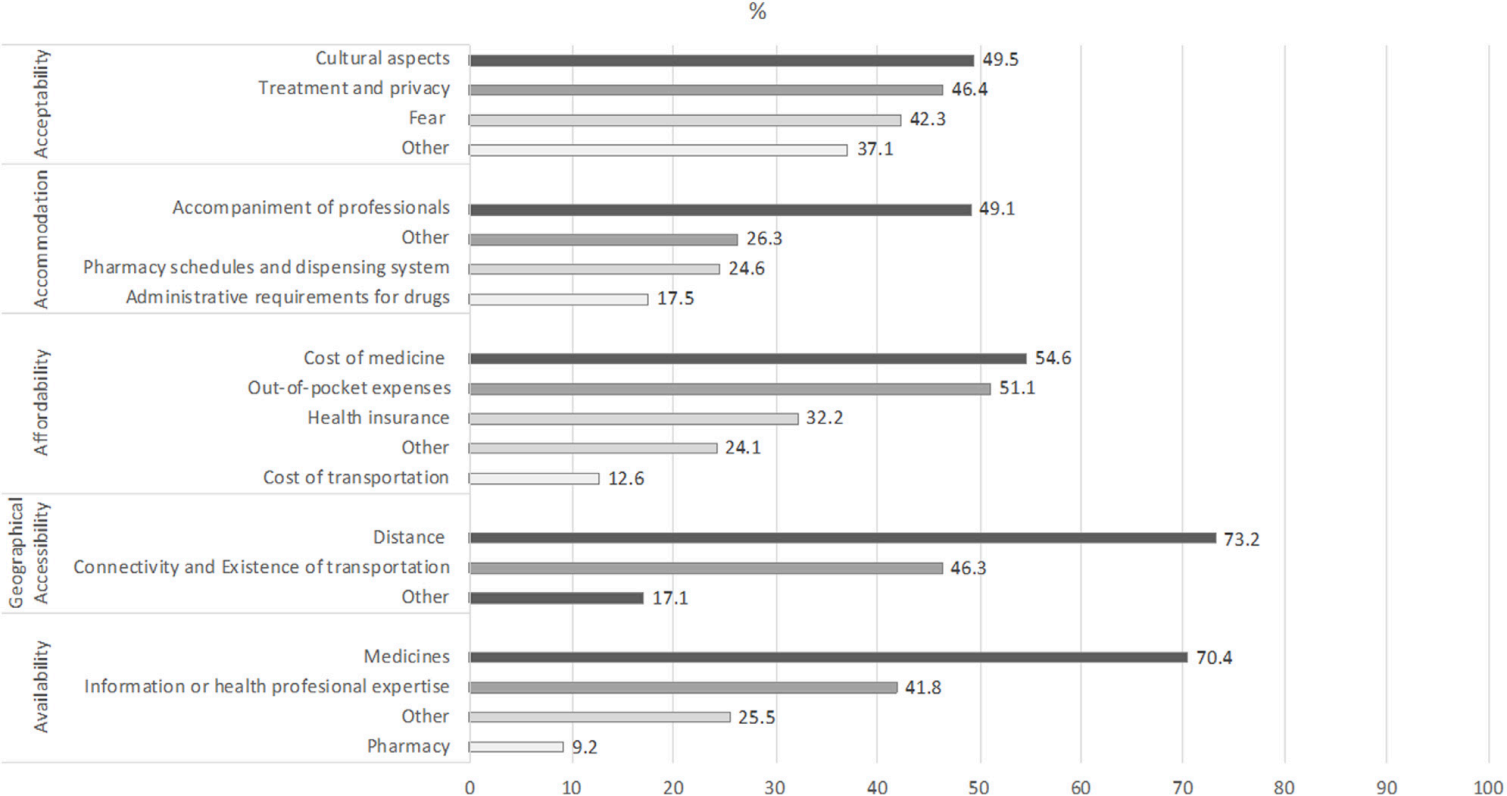
Barriers by sub-dimensions (Chile, 2022). Source: based on [4, 7, 9, 15, 16, 22–24, 26, 27, 35–245].

Of the 219 documents included in the review, reported barriers were identified in: affordability (79.5%, *n* = 174), availability (44.7%, *n* = 98), acceptability (44.3%, *n* = 97), accommodation (25.6%, *n* = 56), and accessibility (18.7%, *n* = 41). Facilitators were identified in: affordability (37%, *n* = 81), acceptability (21.9%, *n* = 48), availability (18.3%, *n* = 40), accommodation (8.7%, *n* = 19), and geographical accessibility (4.6%, *n* = 10). Details of sub-dimensions are found in Figure 3.

Following, the dimensions and their corresponding subdimensions are characterized and defined. Also, Table 2 presents a complementary analysis regarding the settings in which these barriers and facilitators were reported.

**TABLE 2:** Dimensions, barriers and facilitators settings (Chile, 2022).

Dimension	Barrier/facilitators	Setting
Availability	Medicine	There are no major differences according to income in the countries studied. However, barriers stand out in vulnerable contexts such as rural health facilities, remote villages, in public health facilities, in poor urban areas and in poor communities
	Pharmacy	These barriers predominate in UMIC and LMIC (*n* = 5), one was identified in a LIC (Rwanda), and the other three were worldwide
	Information or health professional expertise	Most of these barriers were identified in UMIC and LMIC (*n* = 16); followed by HIC (*n* = 11), especially in the United States (*n* = 8) and finally LIC (*n* = 2), Rwanda and Uganda
	Other	These barriers were identified mainly in UMIC and LMIC (*n* = 9); one in HIC and in LIC (United States and Uganda, respectively)
	Facilitators	Facilitators were identified most in UMIC and LMIC (*n* = 10), followed by HIC (*n* = 4), mostly in the United States (*n* = 3), and one in LIC (Uganda). At all income levels of the countries, training of health professionals, and free and easy access to medicines are relevant. At the global level, the strengthening of national policies, regulation, registration of generics and the drug market are recognized as facilitators
Geographical accessibility	Distance	Most of these barriers were identified in UMIC and LMIC (*n* = 11), followed by HIC (*n* = 6), especially in the United States (*n* = 3)
	Connectivity and existence of transportation	Most of these barriers were identified in UMIC and LMIC (*n* = 8), followed by HIC (*n* = 4) and finally LIC (*n* = 1)
	Other	This was found in HIC such as Singapore, United States and Australia (*n* = 3), followed by UMIC and LMIC (*n* = 2) such as Nepal and South Africa
	Facilitators	Facilitators were identified mainly in HIC (*n* = 5), especially in the United States (*n* = 4), followed by UMIC and LMIC (*n* = 2), India and South Africa. All recognized as facilitators the need for transportation and pharmacies close to the communities (less than 30 min); however, HIC also identified the use of mail order pharmacies
Affordability	Cost of medicines	Although most of the studies are from HIC, and specially the United States (*n* = 25), there is evidence for the cost of medicines as a barrier from LMIC, such as India (*n* = 7) and from UMIC, such as China (*n* = 3)
	Out-of-pocket expenditure	Having to pay for medications is a cross-cutting concern. Although it has been more studied in the context of HIC (19 documents just from the United States), it is also possible to find studies from LMIC, mainly from the north and Sub-Saharan region [e.g., Kenya (*n* = 4); Nigeria (*n* = 3), Ghana (*n* = 3)], and from UMIC, such as Brazil (*n* = 4)
	Cost of transportation	In this case, most of evidence comes from Sub-Saharan Africa (e.g., Kenya, *n* = 3) and South Asia (e.g., India, *n* = 3)
	Lack of financial coverage by the health system or private health insurance	Most of the studies coming from HIC, especially from the United States (*n* = 20)
	Other	Most of the studies from the United States (*n* = 15)
	Facilitators	While most of the studies are from HIC (e.g., United States, *n* = 15) mentioning medication assistance programs or Medicare Part D, there is a growing interest in programs such as “Health has no Price,” in Brazil (*n* = 3)
Accommodation	Pharmacy hours and dispensing system	Although two of the studies regarding dispensing system are from the United States, the rest are from LMIC from the Sub-Saharan Africa (Kenya, Nigeria), and UMIC from Latin America (Brazil, Colombia)
	Administrative requirements	All the studies that mentioned administrative requirements as a barrier are from HIC (*n* = 10), with six of them being from the United States (paperwork)
	Accompaniment	In this case all the studies are from HIC (*n* = 12), nine of them from the United States
	Other	Four studies form HIC, four from LMIC (all from South Asia) and three from UMIC [South Africa (*n* = 2) and Malaysia (*n* = 1)]
	Facilitators	Among evidence from high-income countries, five studies from the US present facilitators such as prescribing clinicians that considers patients’ preferences, translation services, services targeted to specific minority populations, while three studies from Canada mentioned the availability of advice over the phone (or by email), up to date staff, staff that provides support beyond the technical expertise, and interprofessional collaboration
Acceptability	Cultural aspects	This type of barrier is mainly mentioned in HIC (*n* = 25), particularly in the United States (*n* = 21) where language barriers, and ethnic and racial disparities predominate. In UMIC (two studies in Malaysia and two in South Africa) and LMIC (four studies in: India, Bangladesh, Tunisia, and Tanzania), the use of alternative medicines instead of traditional ones is observed
	Fear	Most of the studies mentioning this barrier come from HIC (*n* = 20), mainly the United States (*n* = 19), but the same fears are also identified in UMIC (*n* = 4), in LMIC (*n* = 4) and worldwide (*n* = 13)
	Treatment and privacy	Considerations on the quantity and way of taking medications appear in studies from HIC (*n* = 19), UMIC (*n* = 5) and LMIC (*n* = 7)
		Evidence regarding lack of trust and dissatisfaction with the treatment, as well as the perception of a lower quality of care in public services compared to private ones comes mainly from HIC
	Other	These types of barriers are observed in countries with different income levels; however, most of the studies come from HIC (*n* = 17)
	Facilitators	Although most studies mentioning facilitators come from HIC (*n* = 19), the use of modern devices, such as insulin pens, or the combination of drugs that decrease intake are also identified in UMIC (*n* = 5) and LMIC (*n* = 9). In particular, bilingual care is highlighted in the United States
Other	Other barriers	Most of the studies that mention these barriers are from HIC (*n* = 5) or include countries from different socioeconomic levels (*n* = 5)
	Other facilitators	They are mentioned only in three studies (one in the United States and two worldwide)

Source: based on [4, 7, 9, 15, 16, 22–24, 26, 27, 35–245].

#### Availability

This dimension considers different aspects related to the physical existence of medications and health professional expertise. It was classified into four areas: 1) medicine, refers to the physical availability of medicines [4, 9, 21, 22, 35–99]. 2) Pharmacy, refers to the availability of medication dispensing establishments [35, 46, 54, 61, 62, 65, 94, 99, 100]. 3) Information or health professional expertise, understood, on one hand, as the information or training that professionals have about the disease and its treatment, which is materialized in the prescription of the medication needed [9, 21, 35, 40, 42, 65, 73, 81, 86, 90, 93, 96, 100–116], as well as the availability of professionals who issue the prescription [22, 35, 36, 41, 45, 51, 61, 72–74, 91, 107, 117–119]. 4) Other, considers regulatory, legal and market aspects, patents, regulatory and drug registration processes [4, 44, 57, 65, 73, 86, 120–125]. This last category also includes aspects related to the availability of supplies for the administration of medications (syringes, needles, or reagent strips); the availability of tests or glucometers [9, 15, 35, 46, 56, 91, 101, 102, 112, 126, 127]; and finally, sub diagnoses [99] and drug transport mechanisms [38, 46].

Facilitators in this dimension are related to the political and health system level, and include national regulations for medication purchasing, the definition of an essential list of medicines, evidence-based national strategies for control of chronic diseases, national drug policies, and the strengthening of health information systems, which make it possible to link health risks with the availability of drugs, efficient procurement, increased national supply of generic drugs, and distribution systems for pharmaceuticals and regular monitoring of medicine stocks [4, 9, 39, 44, 55, 56, 63, 65, 72, 73, 77, 80, 99, 102, 123, 124, 128–130]. Other aspects that facilitate availability are providing education, competencies, and training to health professionals [22, 35, 86, 102, 107, 111, 131–134]; easily-accessible medication dispensing facilities (people’s pharmacies, medicine delivered to home, mobile pharmacies in rural settings) [36, 43, 94, 99, 113, 134–136], and integrated health care programs, which optimize the resources allocated for private health care, to benefit other programs [122, 137]. Finally, medical innovation provides more individualized treatment options and treatments adapted to patients [51, 93, 95, 138].

#### Geographical Accessibility

This relates to geographical distance between medicines and the location of users who need them. Three areas were considered: 1) distance, refers to the physical distance that people must travel to obtain the medication they need [4, 7, 9, 35–37, 42, 45, 47, 50, 56, 64, 65, 69, 73, 76, 81, 86, 87, 91, 110, 122, 139–146]. 2) Connectivity and existence of transportation, related to the quality of roads, routes and difficulties related to inclement weather, presence of rivers, jungle areas or areas under socio-political conflict or occupation [35, 42, 61, 65, 67, 122]; furthermore, in this area, the presence of sufficient means of transportation and stops that cover the areas where people reside are considered [38, 45, 46, 56, 69, 93, 110, 112, 135, 139, 142, 146, 147]. 3) Other, considers age and physical disabilities as barriers to transportation [7, 43, 68, 117, 135, 140, 148, 149].

Facilitators identified in this dimension are related to the positive perception of the geographic accessibility of a public health facility, proximity to public and private health facilities, transportation availability, urban location of the household [9, 38, 69, 142, 146, 150, 151], services such as mobile pharmacy in rural settings, mail-order pharmacy and transportation assistance for patients (e.g., access to a vehicle, public transit discounts or medical transportation services) [99, 146, 151, 152].

#### Affordability

Is the relationship between price of medicines and the ability of patients to pay. This dimension was divided into five areas: 1) costs of medicines, in which the high price of medicines represents a barrier to access [9, 15, 21–23, 25, 35, 36, 43, 44, 47, 50–52, 54, 56, 58, 59, 62, 65, 66, 69, 73, 75, 77, 78, 82, 84, 86, 88, 92, 94, 95, 97, 99, 100, 107–109, 111, 112, 116, 121, 122, 124, 126, 128, 140, 142, 145, 147, 153–187], implying the postponement of the treatment initiation, interruption/discontinuity, the use of lower doses or only a part of the prescribed medicines [47, 74, 89, 113, 133, 150, 188–191]. 2) Out-of-pocket expenditure, understood as the direct payment for the medicines [4, 9, 21, 37, 41, 42, 44–46, 49, 51, 63, 65, 67, 68, 70, 71, 79, 86–88, 91, 93, 94, 100, 106, 109, 115, 117, 119, 120, 124, 129, 131, 136, 141, 142, 144, 147, 161, 163, 169, 192–203], which is associated with the ability of households to pay for those medicines; therefore, a lack of money is relevant in this context [43, 60, 69, 96, 146, 172, 186, 204–207], especially considering that paying for medicine can lead households to borrow, sell assets, disregard needs, or even fall into poverty [56, 61, 72, 138, 139, 189, 208, 209]. This goes beyond the price of the medicines or the financial coverage, since even low-cost medicines or a lack of full coverage for very expensive medicines could represent a significant proportion of the expenditure for low-income households, with medicines accounting for a considerable proportion of total health expenditure, both at the household and aggregate levels [7, 35, 47, 50, 53, 55, 62, 73, 99, 121, 122, 128, 157, 162, 168, 210]. 3) Cost of transportation, which considers the cost people must incur to travel to the place where they pick up or purchase their medicine; related to the cost of public or private transportation, and the frequency with which they must assume this cost [22, 25, 35, 36, 38, 42, 43, 47, 48, 50, 56, 62, 65, 72, 88, 96, 112, 122, 135, 144–146]. 4) Lack of financial coverage by the health system or private health insurance, which implies that the person must cover the full cost of the required medicines [7, 9, 15, 22, 25, 26, 35, 42, 43, 54, 57, 62, 64, 65, 69, 72, 81, 86, 99, 117, 119–122, 134, 136, 139, 141, 143–145, 157, 158, 160, 161, 164, 166–169, 172, 180, 186, 189, 199, 205, 209–218]. 5) Other considers: cost of elements required for administering the prescribed medicine (e.g., glucometers, strips, syringes) [56, 63, 74, 93, 127, 132, 219], lack of drug price regulation policies and inefficient purchasing by the public sector [35, 66, 97, 99, 112, 128], cost of consultation or check-up with doctors and specialists [86, 91, 113, 171, 191, 213], cost of travel time [36, 96], sociodemographic characteristics that make it more difficult to afford medications (socioeconomic status, unemployment, age, disability, ethnicity, food insecurity) [65, 115, 122, 133, 135, 140, 172, 180, 186, 195, 202, 212], among others [9, 58, 105, 121, 149, 166, 218, 220, 221].

Regarding facilitators, the following were identified: the provision of free-of-charge medicine and health insurance coverage for pharmaceuticals [7, 21, 35–37, 43, 45–47, 49, 50, 60, 66, 69, 72, 73, 75, 87, 97, 107, 112, 117, 119, 134, 139, 140, 142, 150, 154, 158, 161, 164, 177, 180, 181, 186, 191, 194, 201, 202, 204, 205, 208, 209, 222–225]; health policies such as efficiency in government procurement, price regulation, financial coverage of essential medicines, promoting the use of generic, reducing import duties and promoting local production [4, 44, 55, 56, 58, 59, 65, 99, 120, 122, 124, 137, 157, 162, 189]; global or regional initiatives such as the HEARTS Initiative, the Strategic Fund, WHO’s Model list of essential medicines [9, 65, 123, 129, 149]; among others [22, 71, 74, 82, 95, 96, 113, 121, 144, 169, 175, 188, 207, 226].

#### Accommodation

This refers to the relationship between how resources for delivering medicines to users are assigned and the users’ capability to adapt to these factors. It was divided into four areas: 1) pharmacy hours and dispensing system, considers scheduling issues (e.g., due to shortages or only 1 day per month) [46, 87], inconvenient operating hours (for instance, needing to depend on others or missing working time) [42, 48, 93, 139, 191, 227], inefficient dispensing processes (e.g., monthly prescribing instead of considering longer periods) [42, 110], and long wait times to collect the medicines [60, 71, 93, 191, 227]. 2) Administrative requirements, includes excessive paperwork needed for prescribing certain drugs (newer or more expensive) [110, 166, 215], confusion about how the system works [147], bureaucratic process for obtaining drugs or applying for coverage programs [79, 110, 228], among others [165, 229]. 3) Accompaniment area, considers the lack of support and communication from professionals towards to patients in their treatments [81, 91, 98, 102, 104, 113, 139, 159, 180, 187, 202, 206, 230], very short consultations (excessive workload, lack of adequate time to educate, answer questions, adjust doses, discuss possible adverse effects and the risks of not controlling their condition, inadequate counseling or lack thereof) [22, 40, 42, 74, 86, 103, 127, 159, 187, 202, 231, 232], clinical inertia (follow-up and treatment adjustment) [103, 159], lack of alignment in the prescribing behavior at the same center [102], lack of communication between health staff members (ambiguity regarding responsibilities, no team approach to care) [40, 145, 159], lack of concern by physicians about the cost of treatment [189], negative attitude or mistreatment by staff [71, 139], lack of continuity with the same health care professional [40, 213], and infrequent follow-up/monitoring [42, 127, 133, 179, 206]. 4) Other considers: inadequate staff incentives and training [35, 102, 133, 145], inadequate logistic systems [39], incorrect prescriptions or dispensing [128], legibility of prescriptions [43], inadequate infrastructure [101, 139, 233], long wait times to see the doctor [233], lack of coordination between different providers (e.g., public and private) [37, 145], lack of active community outreach for chronic patients [36, 159], inadequate information technologies (e.g., poor record-keeping) [145, 159], lack of recognition of circular migration [38], and different prescribing behavior of professionals depending on the size of the facility [221].

Among facilitators identified are: the development of multidisciplinary teams [103, 234], mentorship programs for primary care physicians [103], the expansion of nurse prescriber or manager role [22, 81, 103] (like in Vietnam), physicians effectively communicating their knowledge and addressing patient preferences for information [7, 84, 131, 156], patients directed to mail-order pharmacy services [150], community outreach [150], support programs (e.g., over the phone, by email, through disease associations) or medication counseling [35, 112, 131, 150], periodic reevaluation of medication efficacy for patients [218], diverse health care professionals that accommodate patients’ cultural and language background [150, 177], offering community venues to decongest clinics [135] and combining medicine collection with other appointments [38] (like in South Africa), exceptions on the restrictions to treatment duration or dispensing quantities for chronic diseases [35], reorganizing patients flow to facilitate dispensing [35], targeting specific minority populations [199], simplified and harmonized, online prior authorization process [215], logistic improvements [86], and extending clinic hours [42].

### Acceptability

This refers to the degree to which population or specific social groups accept medicines and the factors that increase or decrease the likelihood of their use. This depends both on the attitudes of users towards the characteristics of providers and medicines, as well as on the attitudes of providers towards the characteristics of users. It was divided into four areas: 1) cultural aspects, such as preference for alternative medicines [25, 40, 42, 45, 86, 91, 102, 141, 147, 184, 227], social stigma, sense of failure and psychological resistance to insulin [54, 76, 102, 103, 108, 110, 114, 132, 133, 175, 183, 191, 228, 231, 235–239], language barriers [40, 118, 139, 150, 188, 196, 199, 206], and barriers associated with certain ethnic groups, low socioeconomic level and female gender, among others [9, 26, 118, 139, 140, 142, 150, 156, 171, 180, 200, 218, 240–242], and religious beliefs related to concerns about the purity of insulin [102]. 2) Fear, mainly of the use of injectable insulin (fear of needles), side effects of medications, weight gain and hypoglycemia [4, 15, 25, 40, 54, 68, 74, 84, 89, 98, 102–104, 108, 111, 114, 127, 132, 133, 150, 159, 160, 170, 175, 179, 183, 190, 191, 193, 200, 225, 230, 231, 235–239, 243–245], this fear faced by patients is also reported from the health care providers perspective [40, 103, 104, 114, 231, 238]. 3) Treatment and privacy such as the perception of over-prescription, distrust of patients regarding the correctness of the treatment, its complexity and effectiveness, preference for the use of oral medication and skepticism regarding generic drugs [15, 22, 43, 54, 68, 88, 89, 99, 103, 106, 108, 158, 159, 163, 179, 183, 190, 203, 206, 231, 235, 236, 239, 244, 245]. Furthermore, there is evidence of a lack of trust and dissatisfaction with the treatment provided by health care providers, as well as the perception of a lower quality of care in public services compared to private ones [7, 36, 69, 81, 85, 106, 109, 122, 140, 147, 156, 244]. Health care providers also have negative attitudes towards certain treatments and doubt the efficacy of certain medications and are distrustful of the compliance of patients with treatment [22, 51, 54, 101, 102, 118, 132, 155, 159, 193, 239]. *4*) Other includes lack of knowledge of the disease, illiteracy and low schooling, which affects the understanding of the disease and the need for medication by patients and their families [4, 7, 25, 40, 42, 51, 56, 65, 69, 75, 81, 98, 118, 141, 159, 177, 180, 187, 191, 202, 206, 235]. In this area, the barriers are observed in the denial of the diagnosis [158, 159], the idea that medication is only necessary if the patient is symptomatic [36, 82, 179], and other misconceptions about the disease and its control [46, 61, 68, 84, 91, 106, 110, 180, 218, 236, 237].

Concerning the aspects that facilitate acceptability, one of the most reported refers to educating patients, the community, and schools about the pathologies through structured educational programs, innovative materials, and sustained campaigns [35, 41, 45, 74, 75, 91, 102, 103, 113, 130, 132, 133, 159, 179, 231, 232, 245]. Another relevant aspect is to consider the cultural context of the people and to use an integral approach centered on the patients, based on knowledge, and oriented to the improvement of the patients’ quality of life [63, 65, 85–87, 98, 124, 131, 150, 158, 179, 199, 231, 235, 238, 245]. Concerning this, bilingual health personnel are valued by patients [25, 69, 188, 199]. On the other hand, social and family support also emerges as a facilitator [45, 177], as well as trust in medications and healthcare personnel [7, 154], and some personal, clinical and social background of the patients [69, 141, 179, 208, 232]. Finally, facilitators related to the type of drug and its application include the use of analogs, flexible doses, pen devices, or inhalable insulin (for children and adolescents), as well as the combination of medications in the same drug [9, 35, 74, 99, 103, 132, 133, 160, 163, 174, 175, 231, 237–239]. Moreover, the importance of regulating the information and the marketing of medicines [35], and of focusing messages on the equivalence between brand name products and generics, when the quality of generic medicines is assured [99], are underlined.

### Others

Other barriers that could not be included in the previous dimensions are mainly related to the socioeconomic context of the countries and populations, such as LIC, LMIC and UMIC [35, 55, 63, 109, 120] or poverty [109, 118, 120, 173]. Personal factors such as comorbidities [145], mental health problems [118, 145], disability [118], younger age [26, 140], and lack of time [228] are also included here.

Other facilitators included living in a HIC [55], improved social determinants of health [199], and using disaggregated data for vulnerable groups [35].

## Discussion

Lack of access to medicines for chronic conditions, as the three studied in this review, continues to be a struggle for a significant part of the population worldwide. The present review identified 219 documents that discussed this issue in at least one of its dimensions.

The findings suggest that there are recurrent circumstances that hinder people with the studied conditions, to access their medicines. When analyzing the barriers according to the main dimensions, affordability is the most reported and it is present in all settings described. The most reported barriers were availability of medicines, which is a cross-cutting concern, and long distances to facilities, costs of medicines, accompaniment of professionals, and cultural aspects, most present in HIC, UMIC and LMIC.

Although socioeconomic disparities in access to medicines are present worldwide, especially in vulnerable populations, barriers identified in HIC are mostly related to the health system capacity to cover medicines, which, when segmented, generates, and maintains access gaps in the most vulnerable populations. Also, it seems to be an implicit hierarchical order among the barriers depending on socioeconomic status, were availability and affordability are the first two barriers to overcome, and then the other barriers start to play a role. This can be seen regarding accommodation and acceptability (apart from cultural barriers), that does not emerge as an issue in LIC, as seems to be in HIC.

The results show that the relevance of the barriers is determined by the socioeconomic and cultural conditions in which people live. This finding highlights the importance of the role of health systems in the regulatory and policy context, focusing to meet the needs of their population, and assuring financial coverage and access to free medicines when possible. On the other hand, providers can facilitate access through training their health workers, implementing multidisciplinary approach, bringing medicines closer to the people, having more flexible schedules, and having intercultural translators. At the patient level, health education and disease management are crucial.

Based on the results, three major gaps were identified. First, related to the studied conditions, diabetes was undisputedly the most studied; probably due to the need for positioning new forms of insulin administration [114, 160, 174, 175, 237]. Secondly, regarding the target population, most of the evidence is based on patients rather than providers and health systems. Finally, most of the evidence is from the US, with few specific studies from Latin America, Asia or Africa. This should be considered, as there may be underestimated barriers and facilitators related to the other two pathologies, settings, and actors involved.

The strengths of this study are related, on one hand, to its design, since as a scoping review, different types of barriers and facilitators from various perspectives are systematized, allowing for a broad understanding of the phenomenon. A second strength is the rigor of the methodology based on JBI and PRISMA standards.

However, a limitation of the study is that since reviewing the quality of the evidence is optional in this type of study, low-quality studies that report barriers or facilitators would not be identified. Moreover, as the findings originate from published studies and documents, other characteristics, barriers, and facilitators may exist that are not studied for not being published.

The Tanahasi [16], and Penchansky and Thomas [17] models identify a population that encounters health services and another that remains outside the system. For instance, during the review process, only two articles on diabetic homeless were identified [81, 202]. Future research should consider some groups who are excluded from health services, and who may face different barriers than those identified for the individuals who access them.

## Appendix: Search Strategy

Database: PubMed

Date: 27 January 2020

Key words: pharmaceutical preparations OR drugs OR medicines OR medication

AND

access barriers OR access facilitators OR availability barriers OR availability facilitators OR accessibility barriers OR accessibility facilitators OR accommodation barriers OR accommodation facilitators OR affordability barriers OR affordability facilitators OR cost barriers OR acceptability barriers OR acceptability facilitators

AND

diabetes OR diabetes mellitus OR hypertension OR arterial hypertension OR dyslipidemias OR

hypercholesterolemia OR metabolic syndrome

Filters: humans and languages (English, French, Portuguese, Spanish)

Strategy:

(("pharmaceutical preparations"[MeSH Terms] OR ("pharmaceutical"[All Fields] AND "preparations"[All Fields]) OR "pharmaceutical preparations"[All Fields]) OR ("pharmaceutical preparations"[MeSH Terms] OR ("pharmaceutical"[All Fields] AND "preparations"[All Fields]) OR "pharmaceutical preparations"[All Fields] OR "drugs"[All Fields]) OR "medicines"[All Fields] OR ("pharmaceutical preparations"[MeSH Terms] OR ("pharmaceutical"[All Fields] AND "preparations"[All Fields]) OR "pharmaceutical preparations"[All Fields] OR "medication"[All Fields])) AND ((access[All Fields] AND barriers[All Fields]) OR (access[All Fields] AND facilitators[All Fields]) OR (availability[All Fields] AND barriers[All Fields]) OR (availability[All Fields] AND facilitators[All Fields]) OR (accessibility[All Fields] AND barriers[All Fields]) OR (accessibility[All Fields] AND facilitators[All Fields]) OR "accommodation"[All Fields] AND barriers[All Fields]) OR "accommodation"[All Fields] AND facilitators[All Fields] OR "affordability"[All Fields] AND barriers[All Fields] OR ("costs"[All Fields] AND barriers[All Fields]) OR ("affordability"[All Fields] AND facilitators[All Fields]) OR ("costs"[All Fields] AND barriers[All Fields]) OR (acceptability[All Fields] AND barriers[All Fields]) OR (acceptability[All Fields] AND facilitators[All Fields]) AND ("diabetes mellitus"[MeSH Terms] OR ("diabetes"[All Fields] AND "mellitus"[All Fields]) OR "diabetes mellitus"[All Fields] OR "diabetes"[All Fields] OR ("diabetes mellitus"[MeSH Terms] OR ("diabetes"[All Fields] AND "mellitus"[All Fields]) OR "diabetes mellitus"[All Fields]) OR ("hypertension"[MeSH Terms] OR "hypertension"[All Fields]) OR ("hypertension"[MeSH Terms] OR "hypertension"[All Fields] OR ("arterial"[All Fields] AND "hypertension"[All Fields]) OR "arterial hypertension"[All Fields]) OR ("dyslipidemias"[MeSH Terms] OR "dyslipidemias"[All Fields]) OR ("hypercholesterolaemia"[All Fields] OR "hypercholesterolemia"[MeSH Terms] OR "hypercholesterolemia"[All Fields]) OR ("metabolic syndrome"[MeSH Terms] OR ("metabolic"[All Fields] AND "syndrome"[All Fields]) OR "metabolic syndrome"[All Fields])) AND ("humans"[MeSH Terms] AND (English[lang] OR French[lang] OR Portuguese[lang] OR Spanish[lang]))

The strategy created for PubMed was modified to suit the requirements of the other databases. MeSH terms were only used in the databases and indexes that permit such use.
